# Supplementary material for: The causal relationship between the human gut microbiota and pyogenic arthritis: a Mendelian randomization study
Source: Front Cell Infect Microbiol. 2024 Nov 26;14:1452480. doi: 10.3389/fcimb.2024.1452480 (PMC11629706; doi:10.3389/fcimb.2024.1452480)
Supplement: Supplementary file 1 [file DataSheet1.zip › mendelian test/ebi-a-GCST90027648.csv_forest.pdf]

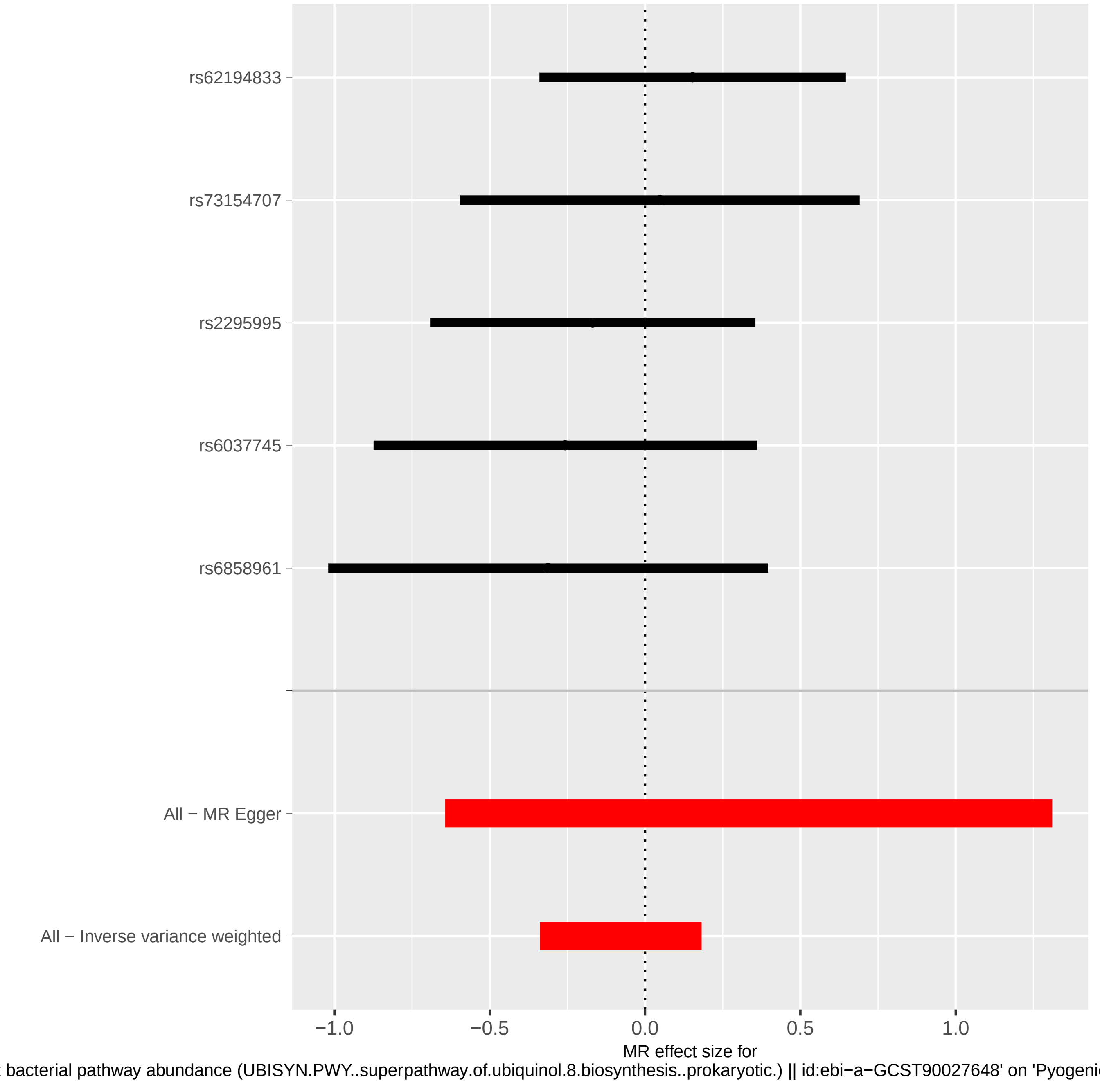
rs62194833
rs73154707
rs2295995
rs6037745
rs6858961
All − MR Egger
All − Inverse variance weighted
−1.0
−0.5
0.0
0.5
1.0
MR effect size for
t bacterial pathway abundance (UBISYN.PWY..superpathway.of.ubiquinol.8.biosynthesis..prokaryotic.) || id:ebi−a−GCST90027648' on 'Pyogenic
